# Beyond drug treatment: a cross-sectional assessment of palliative care services for people living with HIV/AIDS at public health facilities, Abuja, Nigeria

**DOI:** 10.11604/pamj.2021.39.24.23180

**Published:** 2021-05-10

**Authors:** Whenayon Simeon Ajisegiri, Aisha Ahmed Abubakar, Abiodun Egwuenu, Muhammad Shakir Balogun, Elizabeth Adedire, Kabiru Sabitu

**Affiliations:** 1 Nigerian Field Epidemiology and Laboratory Training Programme (NFELTP), Abuja, Nigeria,; 2 Department of Community Medicine, Ahmadu Bello University, Zaria, Nigeria,; 3 Nigeria Centre for Disease Control (NCDC), Abuja, Nigeria

**Keywords:** Palliative care, human immunodeficiency virus, referral service, health facility, people living with HIV/AIDS

## Abstract

**Introduction:**

palliative care offers a care and support system to people living with Human Immunodeficiency Virus (HIV) infection/Acquired Immunodeficiency Syndrome (AIDS). In Nigeria, the palliative care (PC) practice generally is new and still developing. While most studies on HIV/AIDS assess drug treatment and adherence for people living with HIV/AIDS (PLWHA), there is paucity of data on PC services available for them. We therefore assessed the PC services offered and referral services available to PLWHA in health facilities.

**Methods:**

we conducted a cross-sectional study across all public secondary and tertiary health facilities offering HIV care services in Abuja, Nigeria between February and May 2017. We used an interviewer-administered semi-structure questionnaire to collect information from the heads of health facilities. The questionnaire assessed palliative care and referral services for PLWHA. Frequencies and proportions were calculated using Microsoft-Excel.

**Results:**

of the 17 health facilities assessed, only 6 (35.3%) have constituted a palliative care team but only 3 (17.6%) had some sources of fund for PC. Twelve (70.6%) provided nutritional support for PLWHA, 6 (35.3%) provided spiritual and 8 (47.1%) offered bereavement support for families of PLWHA. Sixteen (94.1%) had well-established referral services for PLWHA.

**Conclusion:**

palliative care services for PLWHA were generally poor in all the health facilities. There exists a well-established referral services for PLWHA in most of the health facilities. We recommend that the PC structure for PLWHA should be improved by increasing and ensuring compliance to guidelines and the established referral network should continue to be strengthened.

## Introduction

The prevalence of Human Immunodeficiency Virus (HIV) infection globally is 0.8% with 36.7 million people living with HIV/AIDS (PLWHA) worldwide [1]. Sub-Saharan Africa consists of about 70% of the global population of PLWHA [1]. In 2015, Nigeria was considered to be country with the second highest burden of HIV/AIDS globally with about 228,000 new infection annually, 3.4 million PLWHA [2] and prevalence rate of 3.4% [3]. A recent survey conducted in Nigeria in 2018 showed a decline in these parameters, with annual new infection rates now estimated to have declined to 130,000, 1.9 million PLWHA and national prevalence rate as 1.4% in [4]. The use of antiretroviral drugs prolongs the life of PLWHA. However PLWHA are still predisposed to several chronic conditions, from both the infection and its management [5]. Coupled with Nigeria´s high burden of HIV/AIDS, it is implied that a huge number of PLWHA will require palliative care [6].

“Palliative care is an approach which improves the quality of life of patients and their families facing life-threatening illness, through the prevention, assessment and treatment of pain and other physical, psychosocial and spiritual problems” [7]. It offers a care and support system to both the patients and their relatives [8]. It also includes referring patients to where they could receive social and support services that is not offered in the place of their primary or end-of-life care [9]. Palliative care is therefore necessary in PLWHA, because some patients may present late at the advanced stage of the disease with opportunistic infections, co-morbidities, anti-retroviral therapy toxicity and HIV-associated cancers such as kaposi sarcoma [10-12].

The World Health Organization stated that, based on the nature and burden of HIV and Acquired Immunodeficiency Syndrome (AIDS) in Africa, its palliative care need exceeds that of cancer [13]. In addition, the weak infrastructure of the public health system in sub-Saharan Africa further necessitated palliative care for PLWHA [14]. Despite the fact that significant progress has been made in terms of palliative care in sub-Saharan African countries, only about 5% of those in need of it are actually receiving it [15].

In Nigeria, the practice of palliative care generally is new and still developing [16,17]. Although the national guideline on palliative care for HIV/AIDS outlines the strategies for addressing the multi-dimensional challenges facing PLWHA [8], there is paucity of data on palliative care services provided for PLWHA in the health facilities. We therefore assessed the palliative care services offered to PLWHA in public health facilities and examined the referral services available for HIV patients at the public health facilities.

## Methods

**Study design and area:** we conducted a cross-sectional study across public secondary and tertiary health facilities offering palliative care services in Abuja, Federal Capital Territory (FCT), Nigeria. Abuja is located in the centre of Nigeria with an estimated population of 1,406,239. It is made up of six area councils namely; Abaji, Abuja Municipal, Bwari, Gwagwalada, Kuje and Kwali. There is an estimated total of 293 government hospitals offering various forms of HIV/AIDS services across the six area councils. This comprises of fourteen (14) public secondary and three (3) public tertiary health facilities while others are primary health facilities [18]. We conducted a total population survey of all the 17 secondary and tertiary public health facilities (14 secondary and 3 tertiary) which are distributed in the six area councils as follows: ten in the Abuja Municipal Area Council (AMAC), two in Bwari Area Council, one in Abaji Area Council, one in Gwagwalada, one in Kwali Area Council and two in Kuje Area Council.

**Data collection method and tool:** we used an interviewer-administered semi-structured questionnaire adapted from palliative care literatures [19] and the National Guidelines for HIV/AIDS [8] care to collect data from either the heads of health facility, head of HIV units or designated representative of the various HIV services offered to in-patients and out-patients. The questionnaire assessed: general health care services available for PLWHA, type of palliative care services offered, human resources, technical support, funding level and referral services for PLWHA. Five research assistants with experience in health-related research activities were recruited and trained for a day to ensure standards. All respondents understood and spoke English language and so, there was no need to translate survey instruments to any local language. Advocacy was carried out to the medical directors in the selected health facilities before the dates of interviews. The health facility questionnaires were administered over a three month-period.

**Data analysis:** descriptive statistics was done to describe frequencies and proportions which were displayed in tables and graphs. These variables included general services offered to PLWHA, palliative care structures and services for PLWHA in the 13 public health facilities.

**Ethical consideration:** ethical approval was sought from the Ethics and Research Committee of the Federal Capital Authority Administration and the Health Facilities (FHREC/2016/01/92/24-11-16). Informed written consent was obtained from each hospital´s respondents before administering questionnaire. Anonymity and confidentiality of all health facilities and their respondents was maintained all through the process of the research.

## Results

**HIV care services offered to PLWHA in public health facilities:** all the health facilities, 17 (100.0%) offered HIV counselling and testing as well as antiretroviral therapy (ART) initiation and monitoring, 16 (94.1%) offered management for opportunistic infections, 15 (88.2%) offered prevention-of-mother-to-child transmission services while 10 (66.7%) had information system for ART management. For preventive services, 14 (82.4%) of the facilities offered post-exposure prophylaxis for occupational exposure to HIV while only 12 (70.6%) offered Isoniazide preventive therapy for PLWHA (Table 1).

**Table 1:** HIV care services offered to PLWHA in public health facilities, Abuja, Nigeria - May 2017

Services	Frequency (n=17)	Percentage (%)
**Care and treatment services offered**		
HIV counselling and testing	17	100.0
Partner testing	15	88.2
Barrier contraceptives	14	82.4
Prevention of mother-to-child transmission	15	88.2
Anti-retroviral therapy initiation and monitoring	17	100.0
Management of common opportunistic infections	16	94.1
Nutritional support	13	76.5
Adherence counselling	15	88.2
ART management information system	12	70.6
**Preventive services**		
Post-exposure prophylaxis for occupational exposure	14	82.4
Post-sexual assault exposure prophylaxis	15	88.2
Isoniazide preventive therapy	12	70.6
Cotrimoxazole preventive therapy	14	82.4
Sexually transmitted infections prevention	16	94.1
**Laboratory services**		
Rapid test kits	16	94.1
Enzyme-linked immunoassay (ELISA)	11	64.7
Western blot	6	35.3
Indirect immunofluorescent assays	7	41.2
HIV DNA polymerase chain reaction (PCR)	7	41.2
Reverse transcriptase polymerase chain reaction	9	52.9
Viral load estimation	10	58.8
CD4+ count estimation	15	88.2
Hepatitis screening	16	94.1
**Monitoring of ARV toxicity**		
Liver function test	17	100.0
Electrolyte, urea and creatinine	17	100.0
Full blood count	17	100.0
Lipid profile	17	100.0

**Figure 1:**
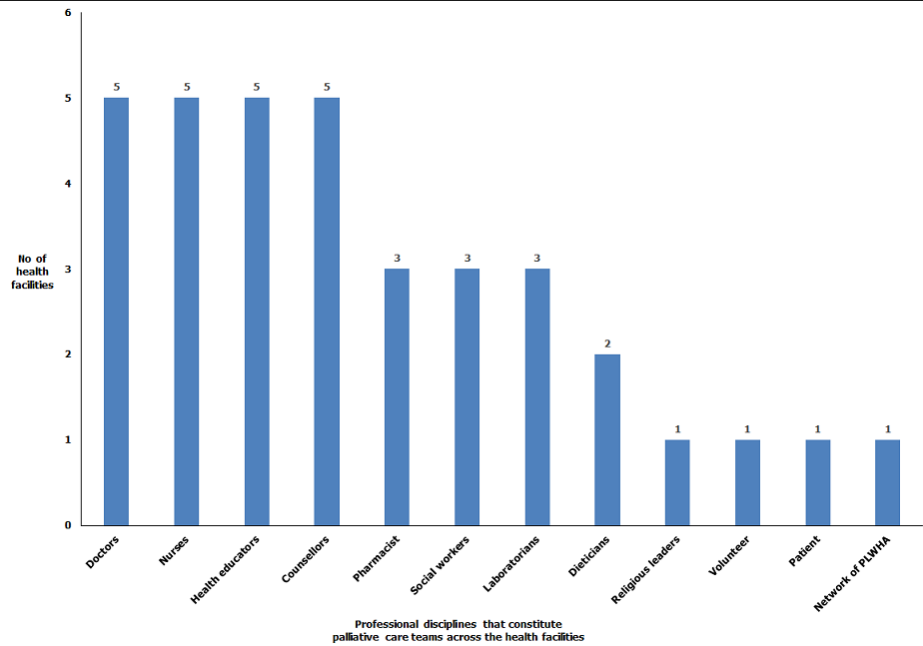
professional disciplines that are members of formally constituted palliative care team for PLWHA in public secondary and tertiary health facilities, FCT, Abuja, May 2017

**Palliative care structure and pain management system for PLWHA:** only 6 (35.3%) of the health facilities had formally constituted palliative care team, 7 (41.2%) of them had some form of training structures as well as guidelines/protocols for palliative care. Only 3 (17.6%) had a dedicated source of fund from palliative care. Eight (47.1%) of the health facilities had a pain management protocol, 7 (41.2%) used standardized pain assessment scale in examining patients‘ pain level and 9 (52.9%) prescribed analgesics based on WHO analgesics ladder (Table 2). Of the six facilities with formal palliative care team, doctors, nurses, health educators and counsellors each were the most commonly mentioned members of the constituted PC team (Figure 1).

**Table 2:** palliative care structure and pain management system for PLWHA in public health facilities, Abuja, Nigeria - May 2017

Variable	Frequency (n=17)	Percentage (%)
**Palliative care structure**		
Facilities with constituted/designated palliative care team	6	35.3
Facilities with training structure for palliative care team	7	41.2
Facilities with some form of guideline/protocol available for palliative care	7	41.2
Facilities with some dedicated funding for palliative care	3	17.6
Sources funding for palliative care (n=3)		
Developmental partners/individual donor	3	100.0
Annual budget	2	66.7
**Pain management system**		
Use of standardized pain assessment scale in examining patients' level of pain	7	41.2
Use of pain management protocol	8	47.1
Prescription of analgesics according to WHO analgesic ladder	9	52.9
Presence of opiate (such as morphine) on essential drug list	7	41.2
Availability of policy on opiate use (e.g. Morphine)	7	41.2

**Palliative care and referral services for PLWHA:** various forms of nutritional support are offered to PLWHA in 12 (70.6%) of the facilities while 11 (64.7%) of them referred patients out to centres where they can access nutritional support. Care for family members of PLWHA were offered by 15 (88.2%) facilities, 9 (52.9%) of the facilities conducted basic skill transfer for family members to support PLWHA while only 7 (41.2%) offered bereavement support to family members of PLWHA. Sixteen (94.1%) of the health facilities had established referral services for PLWHA to ensure the continuum of care. Of these 16 facilities, 13 (81.3%) had a referral network, 9 (56.3%) mentioned that there was a coordinating agency for the referral services and 8 (50.0%) had a designated focal person in charge of the referrals (Table 3).

**Table 3:** palliative care and referral services for PLWHA in public health facilities, FCT, Abuja, Nigeria - May 2017

Services offered by health facilities	Frequency (n=17)	Percentage (%)
**Nutritional support**		
Nutritional support to PLWHA within the health facility	12	70.6
Referral for PLWHA to other centres that offer nutritional supports	11	64.7
**Family support**		
Care for family members of PLWHA	15	88.2
Skill transfer on basic care for family members of PLWHA	9	52.9
**Spiritual support**		
Spiritual support to PLWHA within your facility	6	35.3
Referral of PLWHA for spiritual supports	7	41.2
**Emotional support**		
Bereavement support to family of PLWHA	8	47.1
**Social support**		
Linkage to legal services for PLWHA	4	23.5
Assistance for PLWHA to secure government services such as housing or healthcare	3	17.6
Linkages to food support or income-generating activities	6	35.3
**Availability of referral services for PLWHA**	16	94.1
**Components of the referral services: (N=16)**		
A referral network	13	81.3
A coordinating agency for referral	9	56.3
A focal person in charge of referral	8	50.0
A directory for referral resources	14	87.5
A standardized referral form	15	93.8
A feedback system to track referrals	13	81.3
Documentation of referrals (e.g. standardized referral registry)	14	87.5

## Discussion

The use of ART has been associated with virologic and immunologic benefit, reduction of opportunistic infections and improvement of quality of life of PLWHA [20,21]. It is also impressive that almost all the facilities were equipped with the capacity to estimate CD4+ cell counts and viral load, as these are parameters for immune function and disease progression which are important for prescribing the appropriate palliative care [22]. The available palliative care services in the health facilities included nutritional, spiritual, emotional, social and family support. A study found that 27% of cancer patients and PLWHA required assistance with finding job, 15% required legal advice and 15% required support for their children [23]. Another study also revealed that financial support and improved communication was needed, along with good symptom control, for quality palliative care [24]. Our study reveals a deficiency in the provision of social support for PLWHA. The health facility could improve the quality of lives of PLWHA by exploring options that could help their psychological and financial needs.

Some facilities offer spiritual support within the hospital premises while other referred PLWHA to other sources where they can be helped spiritually. Variation in this service across health facilities may be a reflection of the attending health workers´ opinion, who may view the condition of PLWHA as mainly medical. Previous study reveals that care delivered to those in need of palliative care is greatly influenced by the religious and spiritual belief of the health professionals [25]. Spiritual support give patients requiring palliative care hope and comfort [26]. It is important that relevant stakeholders recognize the importance of spirituality as a strategy for supporting patients, particularly PLWHA. Few health facilities offered some form of bereavement support to the family members of PLWHA after the patient´s demise. This is a limitation in palliative care services provision as the aim is also to help the patient´s families achieve quality of life [27]. A similar study in Rwanda revealed bereavement support was offered to only 18% of families of PLWHA [28]. A possible reason could be due to staff shortages, leading to an inability to provide such care in the presence of increased workload [28]. There is need for health facility services to establish and strengthen palliative care structures that will improve quality of life of PLWHA and their family members.

Formally constituted or designated palliative care team was not a common finding across the health facilities. This in itself might be the reflection of poorly developed palliative care structure in the country [16]. It is expected that the palliative care team must be multidisciplinary in nature in order to achieve effective service provision [28]. Our study found that doctors, nurses, health educators and counsellors were the most commonly mentioned members of the palliative care team. This composition is more comprehensive than the findings by Coughlan who noted that most palliative care team were composed of doctors and nurses only [29]. In Rwanda, social workers, doctors and nurses were the commonest mentioned members of the palliative care team and over 50% of the health facilities had a constituted team [28]. It is expected that this good mix and established structure will provide a holistic support for PLWHA.

Only few health facilities had pain management protocol, used standardized pain assessment scale in examining patients‘ pain level and prescribed analgesics based on WHO analgesics ladder. The finding from this study is comparable to that from Vietnam where 57% of the respondents indicated that their hospitals had policy on pain relief and opioid use [23]. These issues are of great concern as they are associated with under-treatment of pain among PLWHA [30] especially those at the terminal stage of the disease or with co-morbidity who experiences a high level of pain [31]. As the outcome of these will continue to constitute barriers to quality palliative care in the health facilities, it will also impact the quality of life PLWHA. Our study revealed that only few of the health facilities have opiate (such as morphine) included on their essential drug list and also have policy on the use of opiate. The difference in response could also be due to variation in each facility´s adapted drug list since as the only national essential drug list in the country and applicable to all [32]. While the national essential drug list includes opiates, it is not widely available and accessible and this has constituted a major barrier to palliative care [17]. Inadequate or non-availability of essential drugs for pain will limit pain protocol implementation and as well as impact negatively on the palliative care for pain in PLWHA [31].

Newly diagnosed and known PLWHA have unique needs including referral services. These may include referral for emotional distress, mental health disorders or behavioural interventions while for some others and it may be for assistance towards securing employment [33]. Almost all the health facilities have established referral services for PLWHA to ensure the continuum of care. As systemic factors have been identified as the main reasons for ineffective referral system [34], documented findings of the available referral system across the health facilities could be said to be robust. The availability of such a good referral system will reduce delay in seeking care, ensure access to care and monitoring of PLWHA [35]. Those who are lost to follow up can also be easily detected ant tracked appropriately. With a good feedback system in place, collaboration between the referral centres could also be strengthened thereby improving patient care. This robust referral system could be due to the extensive supports that have been given to the HIV/AIDS programme by both the government and partners over the years.

A potential limitation of this study is that responses obtained from the various facilities were self-reported and information were not independently validated by the researchers. However, we made all possible efforts to collect quality and reliable data by obtaining information from key informants, including heads of facilities where possible. This inclusiveness of all public secondary and tertiary health facilities across the whole state makes it representative and gives strength to the study.

## Conclusion

Though there exists a well-established referral linkage for PLWHA in most of the health facilities, palliative care services for this population were generally poor across all the facilities. This will negatively impact the quality of life for PLWHA and their family members. We therefore recommend that the government should improve the palliative care structure and services for PLWHA in public health facilities through increased funding and all supportive mechanism to implement standard palliative care guidelines and pain management protocols.

### What is known about this topic

- Much is known about the palliative care services offered mostly to cancer patients and general services offered to PLWHA such as counselling and testing, anti-retroviral therapy and prevention of mother-to-child transmission.

### What this study adds

- As not much is known about palliative care specifically for HIV/AIDS, this study reveals the type palliative care services offered at public healthcare facility level for people living with HIV/AIDS and the extend of which it is being offered;
- The study also reveals that exist in the palliative care services for PLWHA is inadequate at the selected public healthcare facility level due to lack of funds, guidelines and protocol.
